# A Visual Speech Intelligibility Benefit Based on Speech Rhythm

**DOI:** 10.3390/brainsci13060932

**Published:** 2023-06-08

**Authors:** Saya Kawase, Chris Davis, Jeesun Kim

**Affiliations:** The MARCS Institute, Western Sydney University, Penrith, NSW 2751, Australia; saya.kawase@gmail.com (S.K.); chris.davis@westernsydney.edu.au (C.D.)

**Keywords:** visual speech rhythm, speech perception in noise, foreign-accented speech

## Abstract

This study examined whether visual speech provides speech-rhythm information that perceivers can use in speech perception. This was tested by using speech that naturally varied in the familiarity of its rhythm. Thirty Australian English L1 listeners performed a speech perception in noise task with English sentences produced by three speakers: an English L1 speaker (familiar rhythm); an experienced English L2 speaker who had a weak foreign accent (familiar rhythm), and an inexperienced English L2 speaker who had a strong foreign accent (unfamiliar speech rhythm). The spoken sentences were presented in three conditions: Audio-Only (AO), Audio-Visual with mouth covered (AVm), and Audio-Visual (AV). Speech was best recognized in the AV condition regardless of the degree of foreign accent. However, speech recognition in AVm was better than AO for the speech with no foreign accent and with a weak accent, but not for the speech with a strong accent. A follow-up experiment was conducted that only used the speech with a strong foreign accent, under more audible conditions. The results also showed no difference between the AVm and AO conditions, indicating the null effect was not due to a floor effect. We propose that speech rhythm is conveyed by the motion of the jaw opening and closing, and perceivers use this information to better perceive speech in noise.

## 1. Introduction

It is well established that the information gained by seeing a speaker’s moving face (henceforth ‘visual speech’) benefits speech processing both in quiet and in noise [1,2]. In order to understand the nature of this effect, it is important to be able to examine the speech information provided by different types of speech-related movements, and determine the extent to which perceivers can use such information. In this regard, behavioural research has examined the relationship between properties of the auditory signal and different aspects of visual speech (e.g., how the changes in auditory amplitude associated with narrow focus relate to changes in rigid head motion [3]) and tested whether being presented with such information helps speech perception (e.g., whether seeing rigid head motion facilitates speech perception [4]).

In general, a visual speech recognition benefit is likely based on two overlapping types of speech information: information about what segments have been uttered, and information about speech timing [5]. With regard to pinning down the effects perceiving different types of speech information might have, it is more straightforward to investigate the effects of viewing face and head motion other than the speaker’s mouth. This is because the mouth regions provides both speech-segment and speech-timing information, whereas viewing non-mouth face regions (both peri-oral and more distant regions) likely only provides information about the timing of speech [5]. Evidence for this comes from studies such as Davis and Kim [3], which showed that viewing the speaker’s moving upper head resulted in a small but reliable improvement in speech intelligibility in noise. Likewise, Cvejic et al. [6,7] showed that prosodic contrasts (e.g., narrow focus or questioning) can be distinguished by head motion. The current experiments aimed to further explore the effect that visual speech-timing information has on speech recognition; specifically, to establish whether it is appropriate to attribute any visual influence to a speech rhythm effect.

Many studies have aimed to delineate the effects of speech rhythm on perception by examining auditory speech rhythm. For example, studies have shown that auditory speech rhythm helps listeners perceive speech, especially in difficult listening conditions [8,9]. The beneficial effect of speech rhythm likely occurs because it assists the process of segmentation and decoding [10,11,12], and allows a perceiver to predict and attend to when upcoming speech will occur [11,13]. When speech rhythm is altered or unfamiliar, speech intelligibility is reduced [8,14]. For example, Aubanel et al. [14] tested speech intelligibility for unaltered rhythm and speech that was retimed to change the regularity of timing between syllable onsets or amplitude envelope peaks. It was found that any change in timing resulted in reduced speech intelligibility. A similar effect was found by McAuley et al. [8], who showed that the greater the change in speech timing, the worse the speech recognition in noise. These results suggest that the speech rhythm benefit occurs due to the perceiver’s model of speech rhythm, one that is based on the rhythm characteristics of typically experienced speech.

Very few studies have investigated whether visual speech provides speech-rhythm information that perceivers can use in speech perception. There is, however, good reason to expect that visual speech does impart rhythm information, and that this acts in the same way as auditory speech rhythm. This is because there is a long tradition of viewing speech rhythm as fundamentally arising from speech articulation, which bears on visual speech (since motion related to many aspects of speech articulation can be seen). For example, when it comes to making judgments of speech rhythm (e.g., the isochrony of an utterance) Fowler [15] found that listeners appear to base their judgment on articulatory timing, rather than on the “articulation-free” acoustic information. As to which aspect of articulation is important, MacNeilage and Davis [16] proposed that the basis for speech rhythm is jaw opening and closing. This proposal is consistent with the finding that speech jaw cycles are highly correlated with syllable rate [17], and thus with speech rhythm (given the idea that the syllable has a central position in describing rhythm [18]). From this point of view, the observed motion of the articulators (visual speech) provides a direct source of information about speech rhythm. Indeed, clearly observable peri-oral movements are significantly correlated with fluctuations in the speech envelope at rhythm rates that peak, roughly, at the syllable rate of 3 to 4 Hz [19]. Thus, for current purposes we propose that speech-rhythm information is conveyed by the motion of jaw opening and closing.

**Figure 1:**
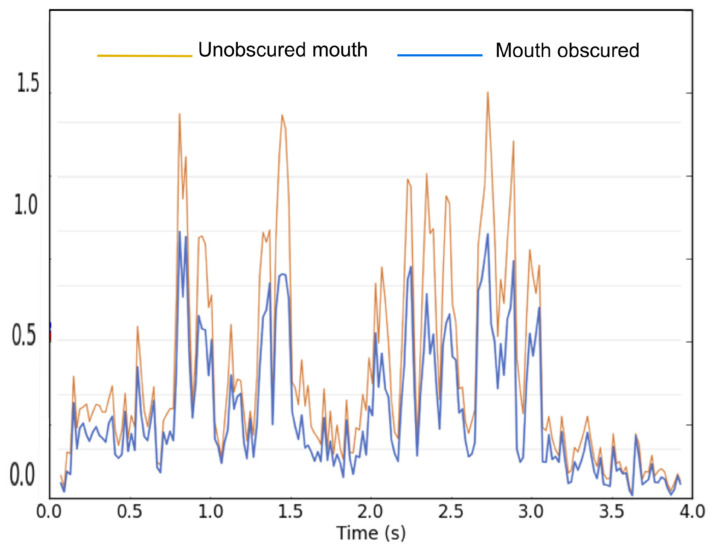
Optic flow magnitude for a sample video with the mouth obscured/unobscured.

Given the central role of visually observable speech articulation as a generator of speech rhythm, it seems plausible that visual speech will provide a perceiver with speech-rhythm information that will in turn help people perceive speech. This was tested in the current study by examining the effect of viewing a speaker’s articulatory movements on speech perception in noise. To isolate visual speech-rhythm information from visual speech-segment information, we used the method developed by Kim and Davis [5]. In their study, the mouth area was obscured by an overlaid opaque circular patch (see Method below). The patch prevents participants from perceiving detailed information about speech segments, but still permits rhythmic articulatory movements to be perceived. Figure 1 shows, using one of the videos in the current study, the magnitude of pixel-based optic flow (as measured by Flow analyzer software [20]) of a speaker’s face with and without the mouth region obscured. The magnitude value is obtained by summing the magnitude components (polar coordinates) of the velocity vectors; thus, the magnitude signal represents the overall amount of motion, ignoring direction. As can be seen, the pattern of motion described by the two curves over time is very similar, with the mouth-obscured curve simply having less overall motion.

Studies using this method (e.g., [5,21]) demonstrated a visual speech facilitation effect, i.e., presenting visual speech, even though it excluded the oral region, facilitated speech recognition in noise (compared to an auditory only condition). What these studies did not do, however, was to probe the nature of this effect. The current set of experiments aimed to do just this, with the specific issue to be investigated being whether or not the facilitation effect is a speech rhythm effect. That is, the research issue was whether seeing the speaker’s peri-oral movements aids intelligibility because it allows the perceiver to tap into speech-rhythm information, which facilitates such things as speech segmentation and decoding. Or it acts as a cue to auditory speech properties, regardless of speech rhythm.

To make the latter position clearer, consider the results of a recent study by Wang et al. [22]. This study examined the effect of non-speech visual cues on the intelligibility of speech in noise that had familiar or unfamiliar speech-timing information (rhythm). The unfamiliar speech rhythm consisted of rhythmically hybrid sentences that were created by randomly combining slow, normal, or fast speech rates (as manipulated by a Synchronized Overlap-Add Algorithm). The visual cues consisted of LEDs that flashed for 50 ms at the onset of each syllable. For the baseline cue condition, the LEDs lit up at the start of the sentence and remained on for its entirety. The results showed that, compared to the control cue, the visual syllable onset cue facilitated speech recognition in noise, but this only occurred for the speech that had the non-standard (hybrid) rhythm. Wang et al. suggested that the auditory temporal cues available from familiar speech rhythm rendered the visual cues for the timing of syllables redundant. 

The current experiments probed the nature of the (peri-oral) visual speech effect by presenting familiar and unfamiliar speech rhythms. Here, the rationale was that, similar to auditory speech, visual speech rhythm would only produce a recognition benefit when the rhythm was familiar. There is, however, a problem in producing natural unfamiliar rhythm. While the familiarity of the auditory signal can be relatively easy to manipulate, the visual one is less tractable. For example, one might be tempted to simply edit the speaker video to alter speech timing; however, such an attempt would probably introduce unnatural artefacts. To avoid this, we used the natural variation in speech rhythm that occurs in foreign-accented speech. Foreign-accented speech, while differing in many ways from the norms of native speech, typically has a prominently different rhythm from that of native speech [23]. More specifically, to present visual speech with an unfamiliar rhythm, we used the speech of an L1 Japanese speaker of L2 English that was rated as having a strong foreign accent (see Method, below), and for which we had ascertained that its auditory rhythm differed significantly from English L1 speech [24]. As additional comparisons, we also used speech from a speaker who was rated as only having a weak foreign accent (i.e., their speech rhythm would be more familiar to the English L1 listeners), and L1 English speech (the familiar, customary rhythm for L1 English listeners). 

In summary, the current experiment tested whether seeing the speaker’s perioral movements would improve speech perception in three foreign-accented speech conditions: (English L1, weak foreign-accented, and strong foreign-accented). Two different predictions can be made depending upon the action of the visual cue. If the visual benefit occurs because the visual cue provides speech-rhythm information, then the information would only be effective when this is informed by a previously developed model (i.e., when it has a familiar speech rhythm). That is, the native speech should show the largest visual benefit (as this rhythm conforms to what would be expected); there should be a similar or reduced visual benefit for the weak-accented speech (as this sometimes departs from what would be expected), and the strong-accented speech may receive only a weak or no visual benefit (as the speech rhythm would not be predictable). On the other hand, if all that is needed for the visual benefit to occur is that the visual cue reliably indicates important temporal information in the auditory speech signal, then a visual benefit should be found for both the weak- and strong-accented speech. Moreover, according to [22], the size of the benefit should be inversely proportional to the degree that the rhythm diverges from what is customary. That is, no visual benefit would be expected for the speech of the native speaker (since here the visual information is redundant), some benefit could possibly accrue for the weak-accented speech, and the greatest benefit would occur for the strong-accented speech (as it departs the most from customary speech rhythm). 

## 2. Method

### 2.1. Participants

Participants were thirty-five native Australian English listeners (6 male; M age = 21.6) who were undergraduate students at the University of Western Sydney and recruited via the university’s research participation system. They had self-reported normal hearing and normal or corrected-to-normal vision. None of the participants were familiar with Japanese-accented English. Note: originally 40 participants were recruited; however, data from five participants were excluded due to their language background (simultaneous bilingualism).

### 2.2. Speakers

The materials consisted of 234 IEEE Harvard Sentences that were spoken by two Japanese speakers and one Australian English speaker (all females; M age = 21.6 years, SD = 3.8) who resided in Sydney at the time of recording. The Japanese speakers consisted of one “inexperienced” English speaker (English L2 strong accent) who had lived in Australia for 4.5 months. Fifteen native Australian English listeners rated her English as “strongly foreign-accented” (8.1 out of 9, where 1 = no accent; 9 = strong accent). The second Japanese speaker was an “experienced” English speaker (English L2 weak accent) who had lived in Australia for 12.5 months. Her English was rated as “mildly foreign-accented” (4.7 out of 9). Both Japanese speakers had begun learning English as a foreign language in Japan at approximately age 13 (i.e., late learners of English). The native Australian English speaker (English L1) was monolingual, born and raised in Sydney; and was recruited at the Western Sydney University. All speakers had no history of speech, vision, or hearing problems (self-report).

#### 2.2.1. Stimulus Recording

The Japanese speakers were selected based on the acoustic analyses of a previous study [24]. This study showed that the speech rhythm of the strong foreign-accented English speaker was significantly different to that of the weak foreign-accented English speaker, which showed a native English speech pattern. 

The audio and video recordings were conducted in a sound-treated recording booth. Speakers were asked to keep their facial expressions neutral, and to face the camera. The speakers were instructed to read aloud each sentence in a neutral tone whilst being recorded. For each participant, the full set of sentences were recorded twice, but the first production was used unless errors or disfluencies occurred in this take. 

Each sentence to be spoken was presented on a 17” computer monitor using DMDX software [25]. The videos were recorded using a Sony HXR-NX30P video camera. Separate audio recordings were made using an externally connected lapel microphone (an AT4033a audio-technica microphone) at 44.1 kHz, 16-bit mono.

#### 2.2.2. Stimulus Editing

Praat [26] was used to mix each of the recorded auditory tracks with the speaker’s speech-shaped noise at a signal-to-noise (SNR) ratio of −4 dB. The speech-shaped noise was generated using all the speaker’s productions and was added to the full length of the recorded stimulus track. 

For the video recording of each sentence, the location of the speaker’s lips was ascertained using the Sensarea software package [27], and the videos edited to ensure that the stimulus speaker’s face appeared in the same position across trials. The video was trimmed to show only the lower region of the face (as in [3,21]). The videos were displayed at a screen resolution of 640 × 480 with 32-bit colour (or grayscale for the static face), and for the moving face conditions played at 50 fps.

#### 2.2.3. Stimulus Condition (AV, AVm, AO)

**Figure 2:**
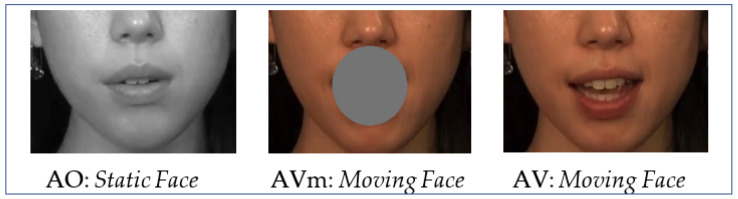
An illustration of the visual stimuli in the Auditory-Only (AO), Audio-Visual (AV), and Audio-Visual with mouth covered (AVm) conditions.

Three types of stimuli were constructed: an AV condition (test trials, *n* = 60, i.e., 20 for each speaker; practice trials, *n* = 18, i.e., 6 for each speaker) where the lower region of the face was presented and the mouth region was visible; an AVm condition (test trials, *n* = 60; practice trials, *n* = 18) where the lower region of the face was presented with mouth motion occluded by a gray circular patch, and an AO condition (test trials, *n* = 60; practice trials, *n* = 18) where only a static grayscale image of the face of the speaker was presented. The size of the occluding patch was selected so that it covered the full extent of mouth articulation. Participants were informed that the grayscale faces would be static so they would not expect these faces to move (see Figure 2). Each condition consisted of the recordings from each of the three speakers (*n* = 20 each), and none of the stimulus sentences were repeated. 

### 2.3. Procedure

Stimulus presentation was carried out via a set of MATLAB scripts based on the Psychtoolbox [28] that also served for response collection. The participants were tested individually in a sound-treated booth. Participants were told that they would see a speaker’s face (either moving or static) while listening over the headphones to speech presented in noise; and that their task was to type what they heard via a keyboard. Several catch-trials were used (*n* = 6 per condition) to encourage the participants to watch the visual stimuli throughout the entire experiment. The catch trials were similar to test trials except that a red cross appeared on the screen during the trial and the participants were instructed not to write down what they had heard but instead were asked to press the “x” key. Any participant who did not respond to the presentation of the “x” catch trials more than two times per condition was excluded from the analyses. Overall, each participant completed all three presentation conditions and was exposed to all three speakers; the presentation order of the presentation conditions and speakers was randomized. In total, the task lasted approximately one hour.

### 2.4. Data Treatment 

The results of the catch-trials indicated that five participants did not pay attention to the visual presentation; hence these participants were excluded and the data reported here are from the remaining 30 participants. Participants wrote down all the words that they heard, However, we scored only open class words (keywords) as closed class words are easy to guess. There were four keywords scored per sentence. For presentation purposes, we summarized the data as proportion of correctly identified keywords. For analysis purposes, for each participant and each item per condition, key words were scored as recognized or not.

The data analyses were conducted in R version 4.1.3 [29]. We fitted a logistic mixed model (glmer, family = binomial) to predict Keywords Correct as a function of Presentation Condition (AO, AVm, AV) and Speaker (English L1, English L2 weak accent, English L2 strong accent), formula: keywords correct~Presentation condition * Speaker. The model included Items and Participants as random effects, formula: list(~1 | Item, ~1 | Participants). The model included random intercepts (for participants and items); however, including random slopes resulted in singular models, so these were not used in the models (see [30,31] on the problems of forcing fits of overparameterized models). The model’s total explanatory power was substantial (conditional R^2^ = 0.45) and the part related only to the fixed effects (marginal R^2^) = 0.18. We used the check_model function from the easystats package, version 0.5.2.5 [32], which indicated model assumptions were met.

## 3. Results

**Figure 3:**
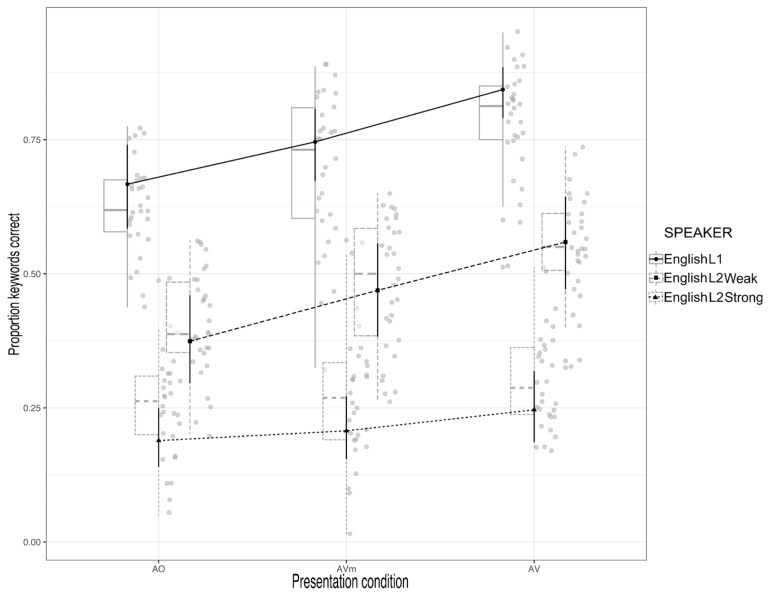
Mean proportion of correct keywords recognized in English L1, English L2 Weak, and English L2 Strong accented speech for the AO, AVm, and AV conditions.

A summary of the data is shown in Figure 3. The figure shows the mean proportion of correctly identified keywords as a function of Presentation Condition and Speaker.

The results of the analysis are shown in Table 1. As can be seen from the table, there was a significant effect of Presentation Condition; a significant effect of Speaker, and a significant interaction between these two main effects. Given these main effects and the significant interaction, further analyses were conducted to examine the possible effects of each of the different speakers.

### 3.1. Planned Contrasts

Planned contrasts, using contrast coding, were carried out with the emmeans package (1.5.1, [33]) and adjusted for multiple comparisons (Holm adjustment). The aim of conducting the contrasts was to determine the statistical significance of the visual rhythm effect (AVm vs. AO), the full visual speech effect (AV vs. AO), and the visual speech-segment effect (AV vs. AVm) for the items of each Speaker. A summary of the results of these contrasts is shown in Table 2. 

As can be seen from the table, for the items spoken by the English L1 speaker, there was a significant effect of visual rhythm, with the proportion of keywords correct being greater in the AVm condition (M = 0.686, SE = 0.013) than in the AO condition (M = 0.616, SE = 0.013). There was also a significant full visual speech effect; the proportion of keywords correct was greater in the AV condition (M = 0.791, SE = 0.011) than in the AO condition (M = 0.616, SE = 0.013). There was also a visual speech-segment effect; the proportion of keywords correct was greater in the AV condition (M = 0.791, SE = 0.011) than in the AVm condition (M = 0.686, SE = 0.013).

The same pattern was found in the recognition of keywords spoken by the English L2 weak foreign-accented speaker, i.e., a significant visual rhythm effect, the proportion of keywords correct being greater in the AVm condition (M = 0.494, SE = 0.014) than in the AO one (M = 0.423, SE = 0.013). There was a significant full visual speech effect; the proportion of keywords correct was greater in the AV condition (M = 0.569, SE = 0.013) than in the AO condition (M = 0.423, SE = 0.013). Finally, there was a significant visual speech-segment effect, with the proportion of keywords correct in the AV condition (M = 0.569, SE = 0.013) being greater than in the AVm one (M = 0.494, SE = 0.014).

A slightly different pattern of effects was found for the items produced by the English L2 speaker with a strong foreign accent. For this condition, there was no significant visual rhythm effect; the proportion of correct keywords in the AVm condition (M = 0.273, SE = 0.013) was not significantly different from that in the AO one (M = 0.256, SE = 0.012). There was a significant full visual speech effect, with more keywords correct in the AV condition (M= 0.309, SE = 0.012) compared to the AO one (M = 0.256, SE = 0.012). There was also a significant visual speech-segment effect, i.e., more keywords were correct in the AV condition (M= 0.569, SE = 0.013) than in the AVm one (M = 0.273, SE = 0.013).

Two final contrasts (Holm adjusted) were conducted. The first was to examine if there was an interaction between size of difference of the AO and AVm conditions (the visual rhythm effect) with the weak foreign-accented and strong foreign-accented speech. This interaction was significant (the visual rhythm effect was larger for the weak foreign-accented English L2 speech), estimate = 0.585 (log odds scale), SE = 0.0974, Z ratio = 6.006, *p* < 0.0001. The last comparison examined whether the size of the speech-segment effect (i.e., AV vs. AVm) differed for the speech of the two L2 speakers. This interaction was not significant, estimate = 0.137 (log odds scale), SE = 0.0974, Z ratio = 1.410, *p* = 0.1584.

### 3.2. Follow-Up Experiment

In Experiment 1, no visual benefit in the AVm compared to the AO condition was found for sentences of the English L2 speaker with a strong foreign accent, whereas there was a benefit for the speech of the weak foreign-accented speaker. This lack of visual benefit for the strong foreign-accented English speech may have been due to its visual speech rhythm being unfamiliar, such that participants could not use rhythm to aid speech recognition. 

However, the lack of a visual benefit may have also been due to a floor effect, i.e., the baseline AO intelligibility was low (compared to the other two speaker conditions), and such a low level of audibility may have resulted in unsuccessful perceptual integration with visual speech information (see [34]). To determine if the latter was the case, we conducted a follow-up experiment in which we aimed to increase the intelligibility of the English L2 strong foreign-accented speech by reducing the level of masking noise used; i.e., adjusting the SNR to +4 dB. If the lack of a visual rhythm effect was due to an inadequate recognition level in the AO condition, then increasing this level should result in a significant visual rhythm effect.

An additional twenty-five native Australian English listeners performed the same perception experiment as in Experiment 1, with only the strong foreign accent stimuli. Data from three participants were excluded due to their language background (i.e., they were simultaneous bilinguals). The data were analyzed using the same GLMM model as Experiment 1. The model’s total explanatory power was reasonable, conditional R^2^ = 0.35.

**Figure 4:**
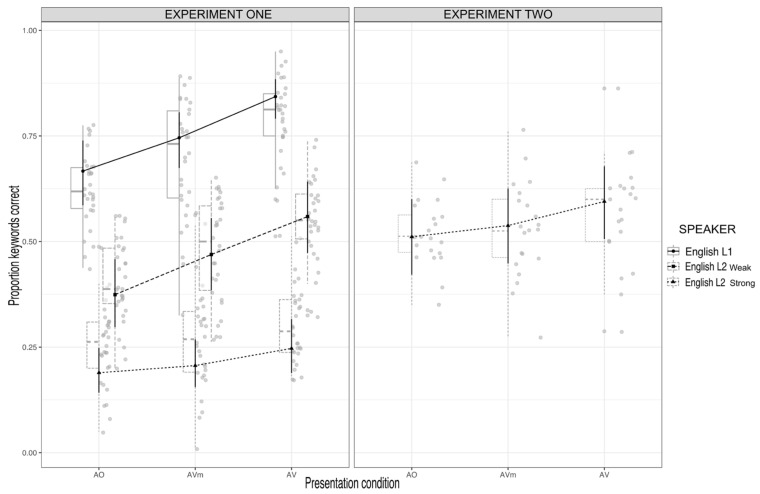
The figure shows the mean proportion of correct keywords for the two experiments. The left-hand panel shows the results for Experiment 1 (for comparison). The right-hand panel shows the results of Experiment 2 for the recognition of weak foreign-accented L2 English speech with a lower noise level (+4 dB). Shown are the AO, AVm, and AV conditions.

Figure 4 shows the results of Experiment 1 and 2 plotted together. As can be seen in the figure (right panel), the manipulation of the masking level had the desired effect of making the strong foreign-accented L2 English speech more intelligible. That is, its mean intelligibility was much higher in Experiment 2 (AO = 0.53, SE = 0.01; AVm = 0.54, SE = 0.01; AV = 0.58, SE = 0.01) compared to Experiment 1 (AO = 0.26, AVm = 0.27, AV = 0.31).

Even though the intelligibility of the strong foreign-accented L2 English speech was improved (indeed, it was even greater than the AO result for the weak foreign-accented L2 English speech), the pattern of results across the presentation conditions was similar to Experiment 1. That is, the difference between recognition scores for the AVm and AO conditions (the visual rhythm effect) was not significant, Z ratio = 1.515, *p* = 0.2840. There was a significant difference between the scores of the AV and AO condition (a full visual speech effect), Z ratio = 4.762, *p* < 0.001. And there was a significant difference in the recognition scores of the AV and AVm conditions (a visual speech-segment effect), Z ratio = 3.276, *p* = 0.003. In sum, given that the increased AO recognition level in Experiment 2, it would seem that the null effect of visual rhythm was not due to a floor effect (see Supplementary Material for all the data reported in the Results).

## 4. Discussion

Previous studies have shown that altering auditory speech rhythm reduces speech recognition in noise [8,14]. This suggests that listeners have modelled the distributional characteristics of the speech rhythm they typically hear, and can use this to facilitate speech recognition (a speech rhythm effect). Of course, when the speech input does not match the expected distribution, recognition performance will suffer. The aim of the current study was to investigate whether any speech recognition benefit that accrues from seeing the peri-oral motion of a speaker would also depend upon the input speech rhythm being familiar.

Two different sets of predictions were made. The first proposed that, because visual speech is directly linked with speech rhythm (via articulation), providing visual speech (even though it excluded the mouth region) would boost speech intelligibility. However, this would only occur when the rhythm was familiar, i.e., just as unfamiliar speech rhythm fails to benefit auditory speech perception, so too would it impact the effectiveness of visual speech. An alternative set of predictions was based on the premise that visual speech simply acts as a cue to when auditory information occurs, which provides a processing benefit. That is, visual (peri-oral) speech would facilitate speech recognition in noise because it acts as a signpost to the auditory signal—one that would be particularly effective for unfamiliar input rhythms. Proposing such a cuing mechanism is not new; for example, a similar idea was put forward to account for the visual speech detection benefit [35]. Moreover, the results of Wang et al. [22] demonstrate that perceivers can use visual cues to facilitate speech processing, and that this is clearest for unfamiliar speech rhythms.

We tested these predictions by using speech that naturally varied in the familiarity of its rhythm. This was achieved by using L2 English speech from two speakers, one with a weak foreign accent and one with a strong foreign accent. Importantly, the rhythm characteristics of the speech of the speaker with the weak foreign accent were more similar to those of L1 English speech than those of the L2 speaker with the strong accent [24]. The results supported the first set of predictions (the rhythm hypothesis) and disconfirmed the second (cuing) hypothesis. That is, there was a significant peri-oral visual effect (significantly more keywords being recognized in the AVm than in the AO condition) for L1 speech, as well as for the speech with the weak foreign accent, but no significant peri-oral visual effect for the speech with a strong foreign accent. In sum, the results suggest that the visual speech intelligibility benefit observed in the AVm is based on speech rhythm. 

Even though there was no visual rhythm effect for the strong foreign-accented L2 English speech, there was a significant speech-segment effect, that is, significantly more keywords were recognized in the AV compared to the AVm condition. Moreover, there was no difference in the size of this effect and the segment effect shown for the speech of the speaker with a weak foreign accent. This result serves to emphasize the likely difference in the way that the visual rhythm effect and the segment one are generated. 

The current results showed an accent familiarity effect, i.e., the visual rhythm effect did not occur for the strong foreign-accented speech but did for the weak accented one. Davis and Kim [4] suggested that visual rhythm information facilitates speech processing because it assists in speech segmentation. The tacit assumption here is that perceivers can use familiar speech rhythm because they have an internal model of the timing of speech based on what they have routinely experienced. To explain the importance of familiarity in the rhythm effect, we propose that this model can be triggered when L2 speech is produced with similar rhythmic timing to that of the L1 speech, and this top-down knowledge supplements bottom-up processing. Indeed, as mentioned, our prior analyses showed that the acoustic timing of the weak foreign-accented L2 speech was similar to the timing of L1 productions. In contrast, the timing of the strong foreign-accented L2 speech was rhythmically less similar to the L1 speech, and so presumably failed to trigger the model.

Having demonstrated a visual speech rhythm effect, we suggest that there are two avenues for future research that could be usefully explored. The first would be to parametrically vary L1 and L2 speech properties to establish the boundary conditions of the visual rhythm effect. This could be achieved by using so-called transplanted speech segments and rhythm stimuli. These auditory stimuli are generated by, for example, retiming the segments produced by an L1 speaker to match the durations of an L2 speaker. These auditory stimuli could then be used to generate visual speech from the latest auditory to visual speech models, which can generalize to the input (see [36]). Experiments could not only test the degree to which speech-segment and speech-rhythm information influence each other, but also test, via repeated exposure, how rapidly new rhythm models can be learned. A second promising avenue for future research would be to use such stimuli in neuroimaging studies. In this way, the brain basis for visual speech rhythm processing could be established, and the general link between visual and auditory rhythm processing explored.

## Figures and Tables

**Table 1:** Summary of the of the results of the generalized linear mixed effects logistic regression analysis of correct Keyword score as a function of Presentation Condition and Speaker (plus the interaction of these effects).

Effect	df	Chi-Sq	*p* Value
Presentation Condition	2,9	294.80	<0.0001
Speaker	2,9	97.44	<0.0001
Presentation Condition × Speaker	4,7	42.74	<0.0001

**Table 2:** Summary of the planned contrasts comparing speech recognition scores for the three different speakers (note: weak and strong refer to foreign accent).

Speaker	Contrast	Estimate	S.E.	Z Ratio	*p* Value
English L1	AVm vs. AO	−0.382	0.0679	5.625	<0.0001
	AV vs. AO	−0.989	0.0721	13.719	<0.0001
	AV vs. AVm	−0.607	0.0732	8.291	<0.0001
English L2 weak	AVm vs. AO	−0.391	0.0658	5.943	<0.0001
	AV vs. AO	−0.752	0.0662	11.356	<0.0001
	AV vs. AVm	−0.361	0.0656	5.502	<0.0001
English L2 strong	AVm vs. AO	−0.115	0.0740	1.552	0.1206
	AV vs. AO	−0.339	0.0729	4.644	<0.0001
	AV vs. AVm	−0.224	0.0720	3.109	0.0038

## Data Availability

The data presented in this study are available in the Supplementary Material.

## Supplementary Materials

The following supporting information can be downloaded at: https://www.mdpi.com/article/10.3390/brainsci13060932/s1.
